# Impact of pneumococcal conjugate vaccines on pneumococcal meningitis cases in France between 2001 and 2014: a time series analysis

**DOI:** 10.1186/s12916-016-0755-7

**Published:** 2016-12-21

**Authors:** Anna Alari, Hélène Chaussade, Matthieu Domenech De Cellès, Lénaig Le Fouler, Emmanuelle Varon, Lulla Opatowski, Didier Guillemot, Laurence Watier

**Affiliations:** 1 Biostatistics, Biomathematics, Pharmacoepidemiology and Infectious Diseases (B2PHI), Inserm, UVSQ, Institut Pasteur, Université Paris-Saclay, Paris, France; 2 Service de Médecine Interne et Maladies Infectieuses, Hôpital Bretonneau CHRU de Tours, Tours, France; 3 National Reference Center for Pneumococci, APHP, Paris, France; 4 Hôpital Européen Georges-Pompidou, Laboratoire de Microbiologie Clinique, APHP, Paris, France; 5 APHP, Hôpital Raymond-Poincaré, Unité Fonctionnelle de Santé Publique (D.G.), Garches, France

**Keywords:** Pneumococcal meningitis, Pneumococcal conjugate vaccine, Surveillance data, Times series modelling, Serotype replacement

## Abstract

**Background:**

Pneumococcal meningitis (PM) is a major invasive pneumococcal disease. Two pneumococcal conjugate vaccines (PCVs) have been introduced in France: PCV7 was recommended in 2003 and replaced in 2010 by PCV13, which has six additional serotypes. The impact of introducing those vaccines on the evolution of PM case numbers and serotype distributions in France from 2001 to 2014 is assessed herein.

**Methods:**

Data on 5166 *Streptococcus pneumoniae* strains isolated from cerebrospinal fluid between 2001 and 2014 in the 22 regions of France were obtained from the National Reference Center for Pneumococci. The effects of the different vaccination campaigns were estimated using time series analyses through autoregressive moving-average models with exogenous variables (“flu-like” syndromes incidence) and intervention functions. Intervention functions used 11 dummy variables representing each post vaccine epidemiological period. The evolution of serotype distributions was assessed for the entire population and the two most exposed age groups (<5 and > 64 years old).

**Results:**

For the first time since PCV7 introduction in 2003, total PM cases decreased significantly after starting PCV13 use: –7.1 (95% CI, –10.85 to –3.35) cases per month during 2013–2014, and was confirmed in children < 5 years old (–3.5; 95% CI, –4.81 to –2.13) and adults > 64 years old (–2.0; 95% CI, –3.36 to –0.57). During 2012–2014, different non-vaccine serotypes emerged: 12F, 24F in the entire population and children, 6C in the elderly; serotypes 3 and 19F persisted in the entire population.

**Conclusions:**

Unlike other European countries, the total PM cases in France declined only after introduction of PCV13. This suggests that vaccine pressure alone does not explain pneumococcal epidemiological changes and that other factors could play a role. Serotype distribution had changed substantially compared to the pre-vaccine era, as in other European countries, but very differently from the US. A highly reactive surveillance system is thus necessary not only to monitor evolutions due to vaccine pressure and to verify the local serotypic appropriateness of new higher-valent pneumococcal vaccines, but also to recognise and prevent unexpected changes due to other internal or external factors.

**Electronic supplementary material:**

The online version of this article (doi:10.1186/s12916-016-0755-7) contains supplementary material, which is available to authorized users.

## Background

*Streptococcus pneumoniae*, a Gram-positive commensal bacterium of the nasopharynx, usually colonises the respiratory tract or nasal cavity. It can cause non-invasive community diseases (i.e. otitis media and sinusitis), invasive pneumococcal diseases (IPDs, i.e. meningitis, bacteraemia) and pneumonia. Before pneumococcal conjugate vaccine (PCV) introduction, the incidence of these IPDs in children aged under 2 years was 44.4/100,000 per year in Europe and 167/100,000 in the United States, and only 11 serotypes caused 70% of them worldwide [1]. In France, the 7‐valent PCV (PCV7, including 4, 6B, 9V, 14, 18C, 19F and 23F serotypes) was introduced and recommended in January 2003. In parallel, a nationwide campaign was initiated to promote better-targeted antibiotic use, which reduced prescriptions by 25% over 5 years [2]. On the one hand, PCV7 led to significantly fewer IPDs due to vaccine serotypes but was associated with more IPDs due to non-vaccine serotypes (especially 19A) because of serotype replacement [3, 4]. On the other hand, the decreased antibiotic use interfered with vaccine-induced serotype replacement by favouring penicillin-susceptible strains (e.g. 7F and 3) probably because of their higher transmissibility and greater invasiveness [5]. The interaction between PCV7 introduction and antibiotic reduction led to important increases of non-vaccine and penicillin-susceptible strains, resulting in an overall rise of the number of pneumococcal meningitis (PM) cases [5]. In other neighbouring countries that introduced PCV7 without lowering antibiotic use, PCV7 introduction had different effects according to the country and the age group: in Germany, Switzerland, England and Wales a substantial reduction in IPDs was achieved [6–8], while in Spain, the impact of PCV7 was attenuated by the increase of non-PCV7 serotypes [9]. Despite these differences, some homogeneity concerning emerging serotypes (19A, 7F, 3) was observed [6, 8, 10, 11]. In the US, overall IPD incidence rates in adults and children declined significantly during the first 3 years of PCV7 use and stabilised thereafter [12, 13]. Nevertheless, PM incidence rates increased among adults, for which non-PCV7-serotype PM increase was greater than PCV7-serotype PM reduction [12].

In July 2010, the French High Council for Public Health recommended replacing PCV7 with the 13-valent PCV13, which covers six additional serotypes (Delta6: 1, 3, 5, 6A, 7F, 19A). To enlarge PCV coverage further, in December 2013, the 23-valent polysaccharide pneumococcal vaccine (PPV23; adding 2, 8, 9N, 10A, 11A, 12F, 15B, 17F, 20, 33F) was also recommended for at-risk children above 6 years old. Its clinical and serologic responses in at-risk children above 2 years old were recently evaluated in the US [14]. A higher valency PCV targeting 15 serotypes (adding 22F and 33F: Delta2) is being evaluated and has shown promising preclinical results but its production is more complex and costs more [15].

Using time-series analyses, we analysed PM trends in France from 2001 to 2014. Differences in PM case numbers were quantified and compared to the pre-vaccine era for each epidemiological period, for the total PM series and by age group and serotype class. Annual numbers and percentages of the main isolated PCV and non-vaccine serotypes for all the periods considered were analysed to study their evolution.

## Methods

### Data sources

PM data for metropolitan France were available from January 2001 to December 2014 from the French National Reference Center for Pneumococci (NRCP), which, since 2001, has received all *S. pneumoniae* strains isolated from cerebrospinal fluid (meningitis) collected through the Observatoires Régionaux du Pneumocoque, a network of about 400 laboratories located in the 13 French regions (67% of public and private French laboratories and around 60% of the French population over the period considered). Serotyping is performed using latex particles sensitised with antisera marketed by the Statens Serum Institute as described in previous publications [4]. Serotypes 6A and 6C were retrospectively identified for the 2001–2009 years using the two new respective factor sera marketed by the Statens Serum Institute in 2010. For each isolate, date of infection, patient’s age, serotype and penicillin G susceptibility, defined by Minimum Inhibitory Concentration (penicillin-non-susceptible is defined as > 0.06 mg/L) were recorded. Because the NRCP database is not exhaustive and to assess the stability of data-system recording, i.e. to ensure that recruitment bias did not evolve over time, we compared NRCP PM data with those provided by Epibac, a non-independent national surveillance network, for which a constant coverage around 74% of the French metropolitan population since 1999 was proved [16]. From Epibac, serotype and penicillin G susceptibility of *S. pneumoniae* strains was not available. The number of “flu-like” syndromes was provided by the French Sentinelles Network (http://sentiweb.org, accessed: 10 October 2015); demographic data were obtained from the French National Institute of Statistics and Economic Studies (http://www.insee.fr, accessed: 2 October 2015). Vaccine coverage was provided by Santé Publique France (http://www.santepubliquefrance.fr/, accessed: 28 September 2016) and community consumption of antibiotics (beta-lactams and macrolides) expressed in Defined Daily Dose (DDD) per 1000 inhabitants and per day were provided by the European Centre for Diseases prevention and Control (http://ecdc.europa.eu/en/Pages/home.aspx, accessed: 28 September 2016).

### Statistical analysis

Temporal variations of PM cases were estimated using time-series analysis through a generalisation of autoregressive–moving-average (ARMA) models (named ARMAX or intervention-transfer–function model), which allows the introduction of input variables. We estimated monthly PM case differences compared to the pre-vaccine era, defined as January 2001–June 2003, as a vaccination effect during the 6 months following PCV introduction was deemed unrealistic because of that lag time. Since PM incidence exhibits seasonality with winter peaks, 12-month periods were defined starting 1 July of year *n* and finishing on 30 June of year *n* + 1, yielding 11 “epidemiological periods” between July 2003 and June 2014. During the baseline period, the monthly number of PM cases expected without any vaccination effect, for a given population size, was estimated. To take into account demographic evolution, the monthly number of PM cases was weighted by the ratio of the mean population size during the baseline period divided by the year *n* population size.

Because of seasonal fluctuations, a trigonometric function had to be estimated and removed to make the series non-seasonal. Assuming that vaccination did not modify seasonal fluctuations, i.e. the post baseline PM series followed the same seasonality as baseline, but affected only the mean, we removed from the entire series (2001–2014) a periodic trigonometric function estimated on the baseline period. We fitted to the non-seasonal series an ARMAX, including 11 dummy variables representing each post vaccine epidemiological period and “flu-like” syndromes incidence, according to previous findings that suggested an IPD–influenza virus interaction [17, 18]. The FLS incidence was added to this model, using a simple transfer function. To check the fitness of the model, we first tested the independence of the residual series (Ljung and Box test) and its Gaussian distribution (Shapiro–Wilk test). The construction and the writing of the model were partly described previously (see Supporting Text S1 in reference [19]).

We quantified the estimated difference in absolute number and percent change of monthly PM cases for each epidemiological period versus the corresponding baseline period, meaning that we calculated the difference between the estimated number predicted by the model and the one expected under the assumption of no change since the baseline period. The same procedure was applied to following PM groups: PCV7-serotype PM cases (4, 6B, 9V, 14, 18C, 19F, 23F), PCV13 Delta6-serotype PM cases (1, 3, 5, 6A, 7F, 19A), other non-vaccine-serotype PM cases and the two most exposed age groups (<5 and > 64 years old).

The evolution of serotype distributions was assessed for the entire population and the two age groups by computing the frequency of each capsular type of *S. pneumoniae* for each epidemiological period.

Analyses were computed with SAS v9.3 (SAS Institute, http://www.sas.com/) and R 3.1.0. All statistical tests were two-tailed and *P* < 0.05 defined significance.

## Results

### Vaccine coverage

**Fig. 1:**
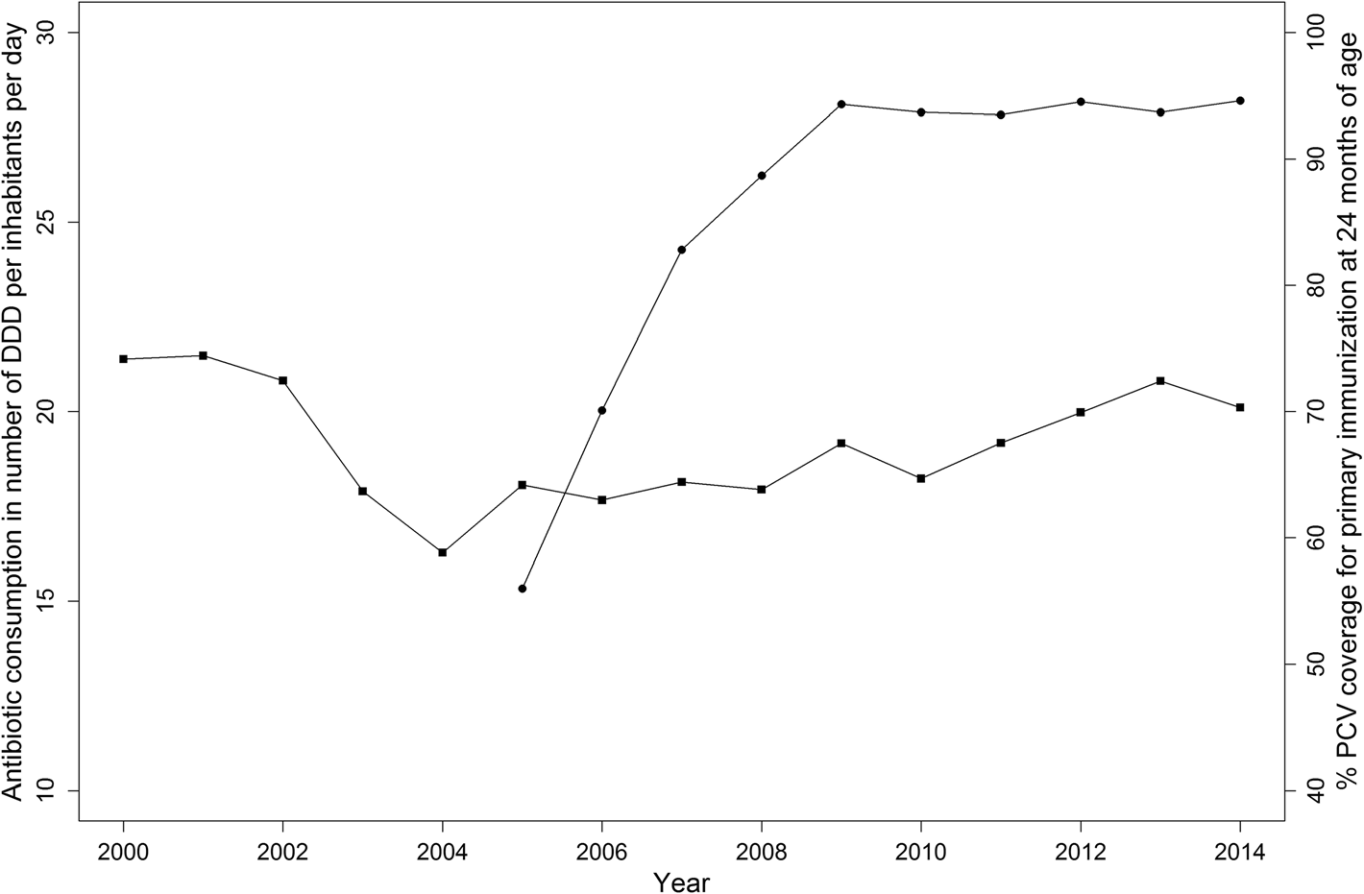
Antibiotic consumption in number of defined daily dose per inhabitants and per day and percentage pneumococcal conjugate vaccine (PCV) coverage. Squares are beta-lactams and macrolides consumption in the community (primary care sector) and dots are PCV coverage percent for primary immunisation at 24 months of age in France from 2000 to 2014

After PCV7 introduction, the vaccine coverage was initially modest (56% of children vaccinated at 24 months in the 2004 birth cohort), but gradually increased to 83% in 2006. The vaccine coverage was 94% when PCV7 was replaced by PCV13 in 2010, and remained stable until the end of the study period (Fig. 1).

### Antibiotic consumption

Community consumption of beta-lactams and macrolides averaged 21.23 DDD per inhabitant per day during the baseline period (2001–2003). The national campaign initiated to promote better-targeted antibiotic use led to a marked decline in the consumption of these two antibiotic classes, with 16.27 DDD per inhabitant per day in 2004. Community consumption then plateaued until 2010, before it started increasing to reach 20.11 DDD per inhabitants per day in 2014 (Fig. 1).

**Fig. 2:**
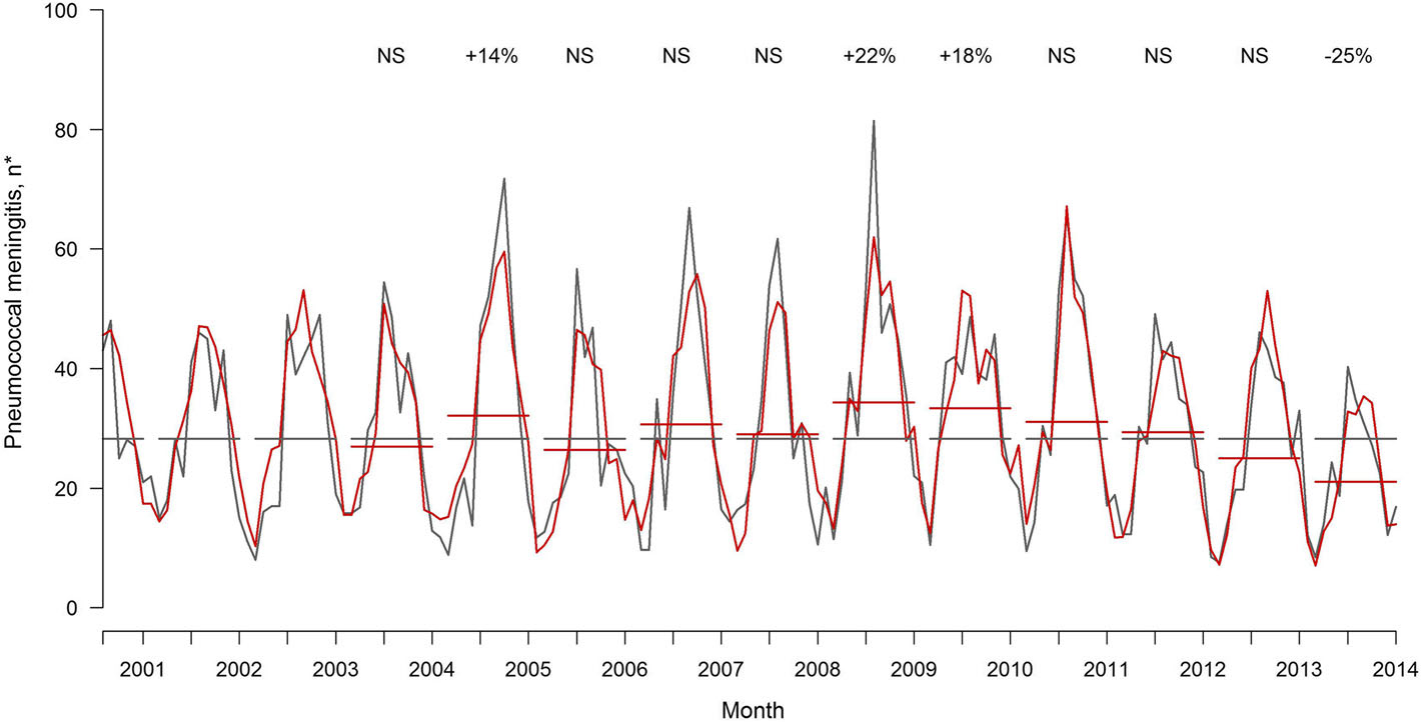
Total monthly pneumococcal meningitis (PM; grey), ARMAX model predictions (red) and estimated percent changes, 2001–2014. Grey lines are the numbers of expected PM cases during epidemiological periods under the assumption of no change compared to the baseline period (2001–2003). Red lines are the model-estimated PM numbers during epidemiological periods. The percentages indicated above the curves are significant relative changes compared to baseline. NS: nonsignificant. *Stable population

Between January 2001 and December 2014, 6025 and 5166 PM cases were recorded in the Epibac and NRCP databases, respectively. In accordance with that stated by Lepoutre et al. [16], comparisons between Epibac and NRCP PM cases enabled us to confidently conduct time-series analyses of NRCP data. Indeed, the comparison with Epibac data indicated stable reporting fidelity of the NRCP surveillance system (regression slope –0.82 (CI), *P* = 0.32). An ARMAX model fulfilling goodness-of-fit criteria was obtained for each series, enabling reliable interpretation of the estimations (Fig. 2, for all serotypes and age series, and Additional file 1: Figure S1 for age-class series).

### PM cases in the entire population

**Fig. 3:**
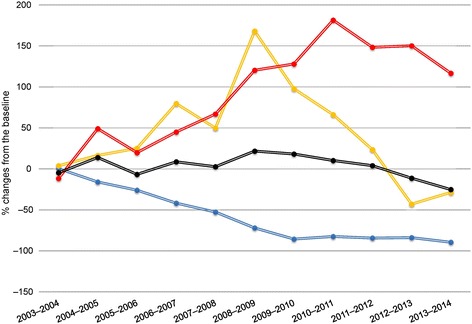
Estimated percent changes compared to the baseline period (2001–2003) during epidemiological periods. Total pneumococcal meningitis cases (black), PCV7 (blue), the six added to PCV7 to obtain PCV13 (yellow) and non-vaccine (red) serotypes

Results for model-estimated deviations are reported in Table 1 and relative PM percent changes in Fig. 3.

**Table 1:** Model-estimated deviations^a^ between numbers of monthly pneumococcal meningitis (PM) cases, according to serotype and compared to baseline

Epidemiological	number of PM cases per month							
Period	PCV7 Serotype	*P* value^b^	Delta6 Serotype^c^	*P* value	Non-vaccine Serotype	*P* value	Total	*P* value
Baseline^d^	15.3	–	5.5	–	7.5	–	28.2	–
2003–2004	–0.1 (–0.66 to 0.78)	0.87	+0.2 (–1.22 to 1.65)	0.77	–0.9 (–2.64 to 0.90)	0.33	–1.3 (–3.87 to 1.22)	0.30
2004–2005	–2.4 (–3.17 to –1.70)	<0.001	+0.9 (–0.61 to 2.41)	0.24	+3.7 (1.88 to 5.50)	<0.001	+3.9 (0.69 to 7.15)	0.017
2005–2006	–4.0 (–4.89 to –3.06)	<0.001	+1.4 (–0.10 to 2.86)	0.07	+1.5 (–0.41 to 3.39)	0.12	–1.9 (–5.36 to 1.62)	0.29
2006–2007	–6.4 (–7.29 to –5.48)	<0.001	+4.4 (2.86 to 5.89)	<0.001	+3.4 (1.50 to 5.27)	<0.001	+2.5 (–1.06 to 5.98)	0.17
2007–2008	–8.0 (–9.01 to –7.09)	<0.001	+2.7 (1.19 to 4.28)	<0.001	+5.0 (3.13 to 6.94)	<0.001	0.7 (–2.82 to 4.33)	0.68
2008–2009	–11.0 (–11.99 to –10.05)	<0.001	+9.2 (7.76 to 10.79)	<0.001	+9.0 (7.11 to 11.00)	<0.001	+6.1 (2.55 to 9.70)	<0.001
2009–2010	–13.1 (–14.11 to –12.09)	<0.001	+5.4 (3.80 to 6.94)	<0.001	+9.6 (7.65 to 11.61)	<0.001	+5.2 (1.47 to 8.79)	0.006
2010–2011	–12.6 (–13.58 to –11.66)	<0.001	+3.6 (2.15 to 5.11)	<0.001	+13.6 (11.74 to 15.56)	<0.001	+2.9 (–0.66 to 6.44)	0.11
2011–2012	–12.9 (–13.88 to –11.94)	<0.001	+1.3 (–0.18 to 2.77)	0.08	+11.2 (9.21 to 13.11)	<0.001	+1.1 (–2.46 to 4.72)	0.53
2012–2013	–12.8 (–13.87 to –11.79)	<0.001	–2.4 (–3.91 to –0.81)	0.003	+11.3 (9.30 to 13.30)	<0.001	–3.2 (–6.85 to 0.47)	0.08
2013–2014	–13.7 (–14.85 to –12.54)	<0.001	–1.6 (–3.08 to –0.06)	0.041	+8.8 (6.68 to 10.88)	<0.001	–7.1 (–10.85 to –3.35)	<0.001

^a^The number of PM cases/month predicted by the model for a fixed population size without vaccination. Estimates were adjusted to flu-like syndrome frequency, for each 12-month period from 1 July to 30 June, followed by their 95% confidence interval

^b^ *P* values are for the comparison of each epidemiologic period’s value versus baseline

^c^Delta6 refers to the six serotypes added to PCV7 to obtain PCV13

^d^Baseline values are expected PM cases per month

#### All serotypes

Total PM numbers were globally stable over time, except for the epidemiological periods in 2008–2009 and 2009–2010, when they increased significantly by 22% (95% CI, 9.0 to 34.4) and 18% (95% CI, 5.2 to 31.2), respectively. Thereafter, PM numbers returned to the baseline level until 2013–2014, when they finally decreased by –7.1 per month (95% CI, –10.8 to –3.35) compared to the pre-vaccine era.

#### PCV7 serotypes

The monthly numbers of PCV7-serotype PM cases declined gradually from 2004–2005 until 2009–2010 (when they reached their maximum diminution of –86% (95% CI, –92.1 to –79.0) from baseline) and remained stable thereafter, at an estimated average of 2 PCV7-PM cases per month.

#### Delta6 serotypes

The number of PM cases due to the PCV13 Delta6 started to increase significantly in 2006–2007, reaching a maximum of almost 15 (95% CI, 13.2 to 16.3) PM cases (baseline + period deviation) per month in 2008–2009, nearly three times the number expected. Their number began to decline the following year, with a statistically significant 43% (95% CI, –71.1 to –14.7) reduction from baseline in 2012–2013 that remained stable in 2013–2014.

#### Non-vaccine serotypes

The monthly numbers of non-vaccine serotype PM cases have risen significantly since 2006–2007. After having increased almost two-fold in 2010–2011 from the baseline, they stabilised thereafter. The 2013–2014 diminution of the increase did not differ significantly from the previous year (+8.8; 95% CI, 6.68 to 10.88 vs. +11.3; 95% CI, 9.30 to 13.30 in 2012–2013).

### PM cases among age groups

#### Children under 5 years old

**Fig. 4:**
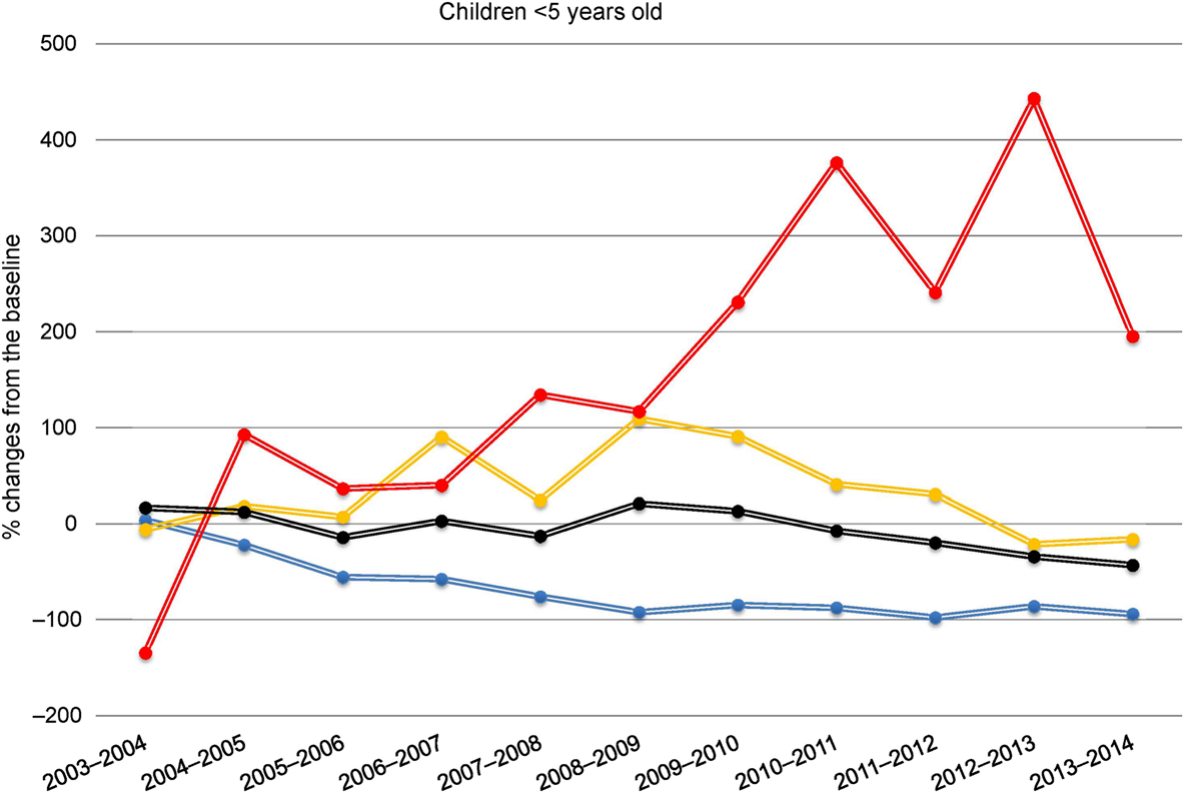
Estimated pneumococcal meningitis (PM) cases percent changes compared to baseline (2001–2003) for children under 5 years old. Total PM cases (black), PCV7 (blue), the six added to PCV7 to obtain PCV13 (yellow) and non-vaccine (red) serotypes

PM cases number trends among children younger than 5 years old were similar to those observed for the overall population. After an estimated rise of almost +2 (95% CI, 0.31 to 3.05) cases/month in 2008–2009, total PM number for children under 5 years started to decrease in 2011–2012 and displayed a reduction of 43% (95% CI, –60.0 to –25.5) in 2013–2014 (Table 2 and Fig. 4). PCV7-serotype PM cases began to decrease in 2004–2005 and they have almost been eradicated since 2007–2008. The number of PCV13-serotype PM cases rose between 2008 and 2010 but decreased thereafter and returned to pre-vaccine era numbers. Non-vaccine serotype PM cases significantly increased since 2007–2008 and until 2012–2013, when they reached their maximum growth of +3.9 PM cases (95% CI, 3.31 to 4.54) per month. This increase was significantly minor in 2013–2014 (+1.7; 95% CI, 1.09 to 2.37 vs. +3.9; 95% CI, 3.31 to 4.54 in 2012–2013, *P* = 0.01).

**Table 2:** Model-estimated deviations^a^ between monthly numbers of pneumococcal meningitis (PM) cases in children under 5 years old, compared to baseline

Epidemiological	Number of PM cases per month							
Period	PCV7 serotype	*P* value^b^	Delta6 serotype^c^	*P* value	Non-vaccine serotype	*P* value	Total	*P* value
Baseline^d^	5.5	–	1.6	–	0.8	–	8.0	–
2003–2004	+0.2 (–0.26 to 0.67)	0.39	–0.1 (–0.76 to 0.56)	0.76	–1.2 (–1.74 to –0.65)	<0.001	+1.3 (–0.02 to 2.70)	0.05
2004–2005	–1.2 (–1.69 to –0.75)	<0.001	+0.3 (–0.36 to 0.97)	0.37	+0.8 (0.23 to 1.42)	0.007	+1.0 (–0.39 to 2.36)	0.16
2005–2006	–3.0 (–3.51 to –2.59)	<0.001	+0.1 (–0.54 to 0.78)	0.72	+0.3 (–0.26 to 0.91)	0.28	–1.1 (–2.47 to 0.20)	0.09
2006–2007	–3.2 (–3.64 to 2.70)	<0.001	+1.5 (0.84 to 2.16)	<0.001	+0.3 (–0.22 to 0.93)	0.23	+0.2 (1.11 to 1.58)	0.73
2007–2008	–4.2 (–4.64 to –3.71)	<0.001	+0.4 (–0.26 to 1.07)	0.23	+1.2 (0.61 to 1.77)	<0.001	–1.0 (–2.38 to 0.32)	0.14
2008–2009	–5.1 (–5.56 to –4.56)	<0.001	+1.8 (1.14 to 2.49)	<0.001	+1.0 (0.42 to 1.65)	<0.001	+1.7 (0.31 to 3.05)	0.016
2009–2010	–4.6 (–5.14 to –4.15)	<0.001	+1.5 (0.82 to 2.19)	<0.001	+2.0 (1.44 to 2.65)	<0.001	+1.0 (–0.34 to 2.45)	0.13
2010–2011	–4.8 (–5.27 to –4.35)	<0.001	+0.7 (0.01 to 1.34)	0.045	+3.3 (2.74 to 3.92)	<0.001	–0.6 (–1.93 to 0.75)	0.38
2011–2012	–5.4 (–5.82 to –4.90)	<0.001	+0.5 (–0.15 to 1.16)	0.13	+2.1 (1.56 to 2.71)	<0.001	–1.6 (–2.92 to –0.26)	0.019
2012–2013	–4.7 (–5.24 to –4.21)	<0.001	–0.3 (–1.04 to 0.33)	0.31	+3.9 (3.31 to 4.54)	<0.001	–2.7 (–4.14 to –1.36)	<0.001
2013–2014	–5.2 (–5.71 to –4.62)	<0.001	–0.3 (–0.93 to 0.39)	0.42	+1.7 (1.09 to 2.37)	<0.001	–3.5 (–4.81 to –2.13)	<0.001

^a^The number of PM cases/month predicted by the model for a fixed population size without vaccination. Estimates were adjusted to flu-like syndrome frequency, for each 12-month period from 1 July to 30 June, followed by their 95% confidence interval

^b^ *P* values are for the comparison of each period’s value versus baseline

^c^Delta6 refers to the six serotypes added to PCV7 to obtain PCV13

^d^Baseline values are the numbers of expected PM cases per month

#### Adults above 64 years old

For total PM cases among individuals above 64 years old, a significant monthly PM reduction of –2.0 (95% CI, –3.36 to –0.57) was estimated in 2013–2014 (Table 3 and Additional file 1: Figure S2). The number of PCV7-serotype PM cases reached its maximum diminution in 2009–2010 (–2.8 PM cases; 95% CI, –3.39 to –2.28). After steadily increasing until 2009–2010, the number of PCV13-serotype PM cases successively declined (–87%; 95% CI, –152.5 to –21.5) since 2012–2013. The non-vaccine serotype PM cases reached their maximum increase in 2011–2012 (+4.5; 95% CI, 3.64 to 5.36) and then stabilised.

**Table 3:** Model-estimated deviations^a^ between monthly numbers of pneumococcal meningitis (PM) cases in adults above 64 years old, compared to baseline

Epidemiological	Number of PM cases per month							
Period	PCV7 serotype	*P* value^b^	Delta6 serotype^c^	*P* value	Non-vaccine serotype	*P* value	Total	*P* value
Baseline^d^	3.7	–	1.3	–	2.0	–	6.7	–
2003–2004	–0.03 (–0.50 to 0.44)	0.89	+0.3 (–0.51 to 1.19)	0.43	+1.2 (0.34 to 2.03)	0.006	–1.2 (–2.60 to 0.18)	0.09
2004–2005	–0.2 (–0.75 to 0.26)	0.35	+0.5 (–0.35 to 1.41)	0.24	+1.8 (0.94 to 2.62)	<0.001	+0.6 (–0.82 to 2.00)	0.41
2005–2006	–0.6 (–1.18 to –0.10)	0.021	+0.5 (–0.30 to 1.40)	0.20	+1.6 (0.73 to 2.44)	<0.001	–0.4 (–1.79 to 1.01)	0.58
2006–2007	–0.1 (–0.68 to 0.40)	0.60	+1.3 (0.49 to 2.19)	0.002	+2.1 (1.23 to 2.93)	<0.001	+1.5 (0.16 to 2.95)	0.03
2007–2008	–0.9 (–1.47 to –0.39)	<0.001	+1.1 (0.28 to 2.00)	0.009	+2.4 (1.50 to 3.23)	<0.001	+0.3 (–1.11 to 1.69)	0.68
2008–2009	–1.5 (–2.06 to –0.93)	<0.001	+3.7 (2.83 to 4.55)	<0.001	+3.3 (2.41 to 4.18)	<0.001	+1.3 (–0.07 to 2.77)	0.06
2009–2010	–2.8 (–3.39 to –2.28)	<0.001	+2.7 (1.81 to 3.56)	<0.001	+3.1 (2.23 to 4.02)	<0.001	+0.3 (–1.14 to 1.71)	0.69
2010–2011	–3.1 (–3.59 to –2.52)	<0.001	+0.7 (–0.18 to 1.54)	0.12	+3.6 (2.74 to 4.45)	<0.001	+0.6 (–0.66 to 6.44)	0.11
2011–2012	–2.2 (–2.78 to –1.73)	<0.001	–0.4 (–1.26 to 0.45)	0.35	+4.5 (3.64 to 5.36)	<0.001	+0.5 (–0.84 to 1.93)	0.43
2012–2013	–2.7 (–3.28 to –2.15)	<0.001	–1.2 (–2.03 to –0.29)	0.009	+3.3 (2.42 to 4.20)	<0.001	+0.5 (–0.92 to 1.94)	0.48
2013–2014	–3.1 (–3.70 to –2.47)	<0.001	–1.1 (–1.96 to –0.25)	0.010	+2.8 (1.98 to 3.73)	<0.001	–2.0 (–3.36 to –0.57)	0.006

^a^ The number of PM cases/month predicted by the model for a fixed population size without vaccination. Estimates were adjusted to flu-like syndrome frequency, for each 12-month period from 1 July to 30 June, followed by their 95% confidence interval

^b^ *P* values are for the comparison of each period’s value versus baseline

^c^ Delta6 refers to the six serotypes added to PCV7 to obtain PCV13

^d^ Baseline values are the numbers of expected PM cases per month

### Change of serotype distributions

**Fig. 5:**
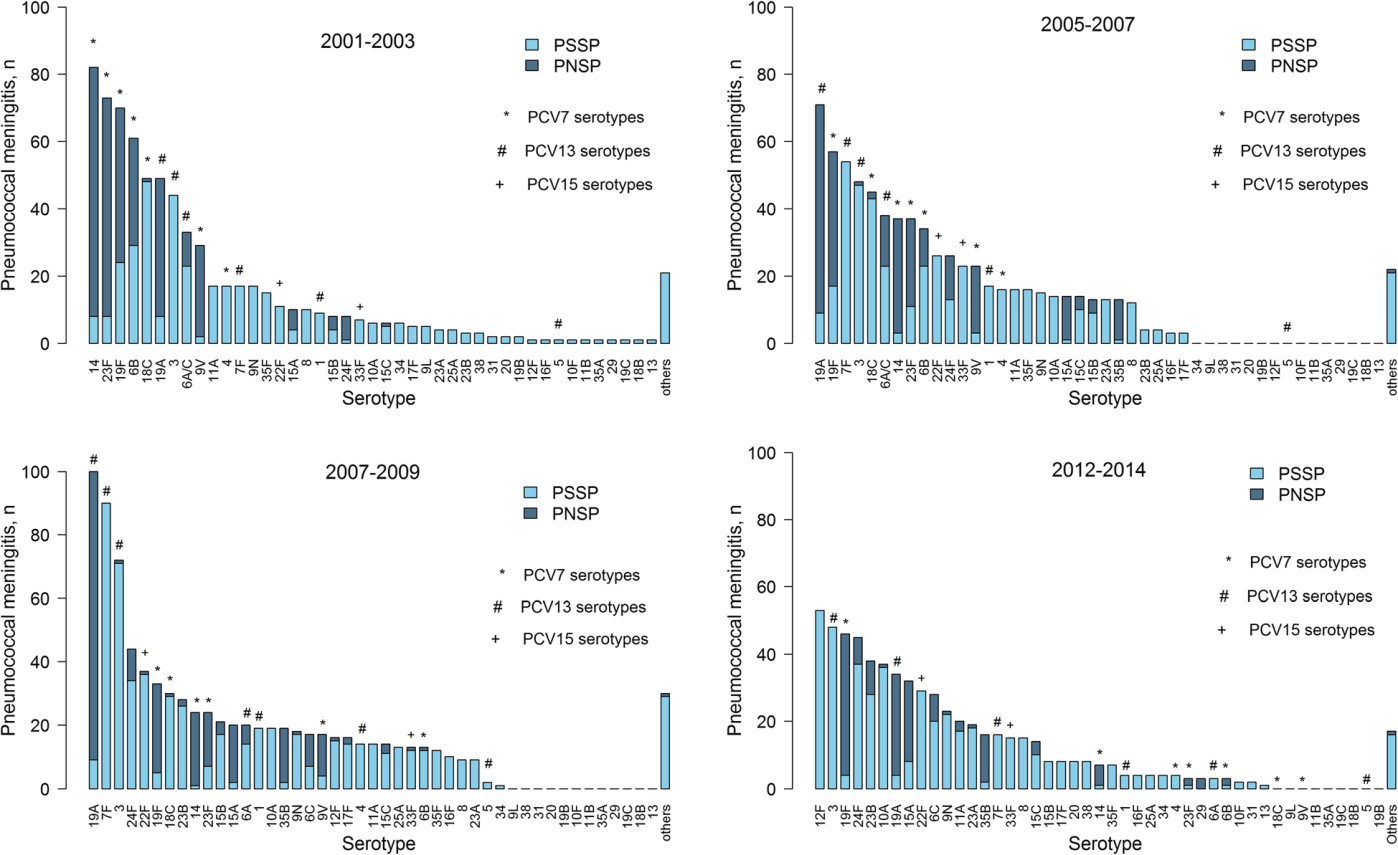
Numbers of pneumococcal meningitis according to serotype and penicillin susceptibility. PSSP denotes penicillin-susceptible *Streptococcus pneumoniae* and PNSP penicillin-non-susceptible *Streptococcus pneumoniae*

Before the vaccine (2001–2003), PCV7 serotypes were the most frequent (53% of total PM cases) and they were principally penicillin-non-susceptible (Fig. 5). After PCV7 introduction, serotypes 19A, 7F and 3 became dominant, while 12F, 23B, 24F and 35B significantly emerged, with rankings increased by at least 10 positions within the serotype pattern, regardless of penicillin susceptibility. In 2012–2014, 4 years after introducing PCV13, other serotypes appeared in the top serotype-distribution ranks. Among non-vaccine serotypes, 12F became dominant (from 2% of PM cases in 2007–2009 to 8%), while the emerging 24F serotype confirmed its position among the most recurrent non-vaccine serotypes, and serotypes 23B, 10A, 15A and 6C clearly became more widespread. The second most common serotypes 3 and 19A, both included in the PCV13, remained relatively high even after its introduction (8% and 6% of the total PM cases, respectively). Surprisingly, PCV7-serotype 19F rose again after its first post-PCV7 reduction and became the third most frequent serotype (and first among the penicillin-resistant serotypes) in 2012–2014. Delta2-serotypes 22F and 33F, which are expected to be added to the future possible PCV15, accounted for only 5% and 2%, respectively, of the total serotypes isolated in 2012–2014. Percentages and numbers of the main serotypes isolated from PM cases are shown in Additional file 1: Table S1.

**Fig. 6:**
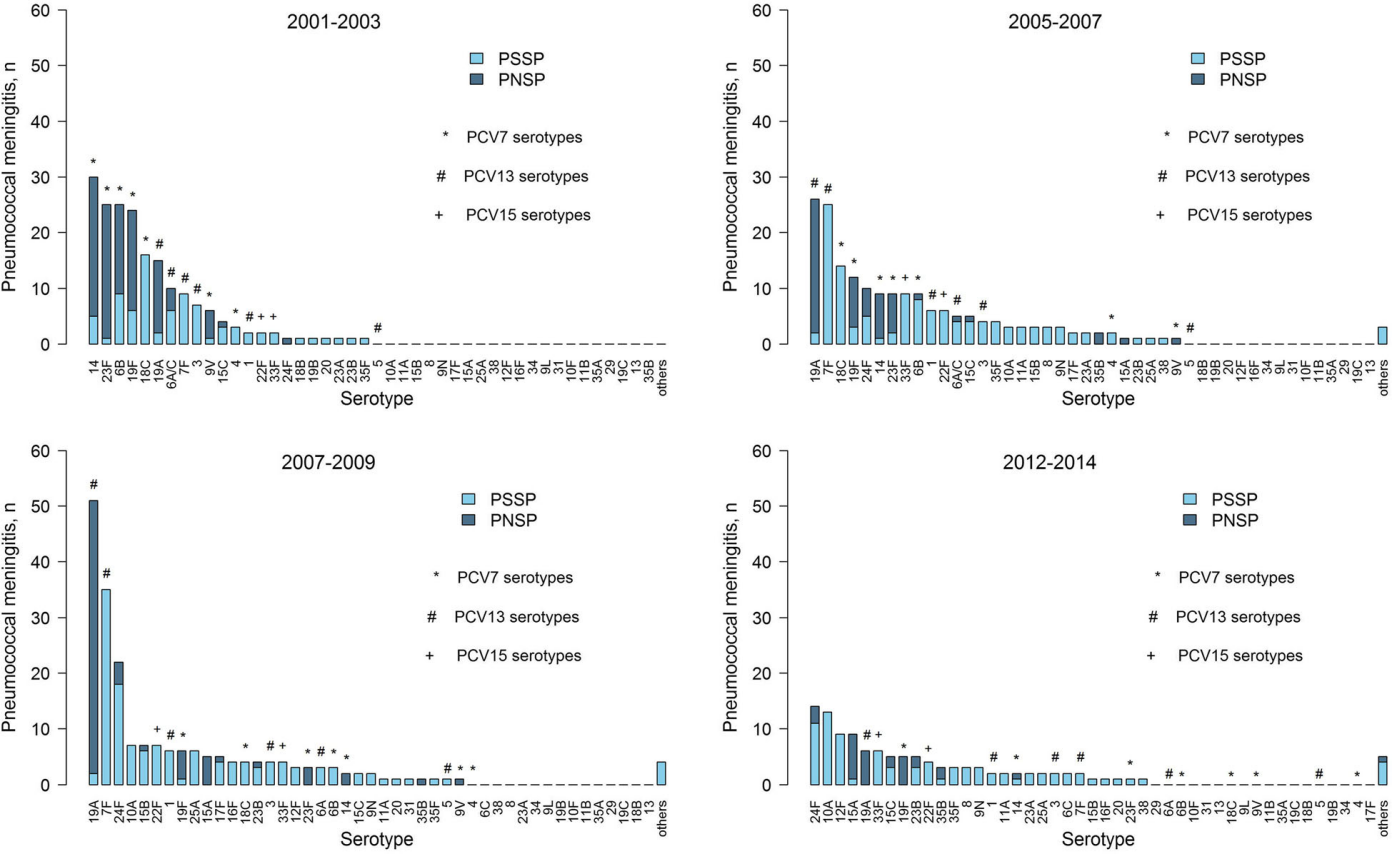
Number of pneumococcal meningitis (PM) cases occurring in children under 5 years old according to serotype and penicillin susceptibility. PSSP denotes penicillin-susceptible *Streptococcus pneumoniae* and PNSP denotes penicillin-resistant *Streptococcus pneumoniae*

For children under 5 years old (Fig. 6), PCV13 introduction led to the remarkable dominance of non-vaccine serotypes, with 24F, 10A, 12F and 15A responsible for approximately 39% of the PM cases in 2012–2014. Vaccine serotype 3 was rare (2%), while the PCV15 Delta2-serotypes 33F and 22F represented 5% and 4%, respectively, of this age group’s total PM cases. For adults above 64 years old (Additional file 1: Figure S3), serotype 6C showed the most remarkable rising to the dominant rank in 2012–2014 (10% of PM cases). Non-vaccine serotypes 23B (9%) and 24F (6%) and vaccine serotypes 19F (8%), 3 (7%) and 7F (6%) were the most frequently observed in this age group during 2012–2014. The PCV15 Delta2-serotypes accounted for 6% (22F) and 3% (33F) of the total PM cases.

## Discussion

The first evident benefit of the cumulative effects of PCV7 and PCV13 on the entire French population appeared 11 years after the former’s introduction, with a significant 25% decrease of the total PM cases. We did not observe population-wide reductions in total PM numbers following PCV7 introduction, although vaccine-specific serotypes did reduce. In contrast, following PCV13 introduction in 2010, a reduction of 25% in all PM numbers in the population was observed in 2013–2014. We confirmed the positive PCV7 impact on young children and its herd-immunity effect on the entire population, as well as partial serotype-replacement phenomena. The significant increase observed in 2008–2009 (caused by nonPCV7 serotypes) for total PM cases suggested that PCV7 benefits were quickly overtaken by serotype replacement, among others (see below). During the following periods, PCV13 effectively offset this augmentation, despite non-vaccine-serotype reinforcement, which counterbalanced and attenuated the PCV13 diminution effect on total PM cases. This decline was already evident in 2011–2012 for children under 5 years, in accordance with previous studies [16, 20]. Furthermore, adults above 64 years old also benefited from herd immunity [21].

The reasons for the long delay between the first PCV introduction and effective reduction of total PM numbers, and why a second vaccine (PCV13) was necessary to remedy the consequences of the first, could be different. When PCV7 was introduced in 2003, the proportion of PM cases caused by its serotypes in the French population was lower than in other countries [22] and its coverage was only 56% in children born in 2004 [16]. In contrast, PCV13 composition included the most virulent serotypes in the postPCV7 period and PCV coverage was above 90%. Other factors, like antibiotic consumption or natural pneumococcal ecologic evolution, could also have had different and unexpected consequences on PM evolution. During the PCV7 immunisation period, antibiotic consumption decreased, whereas it remained stable during PCV13 introduction. These circumstances could explain the prolonged interval required to achieve effective PM diminution.

Although PCV13 use outcome has been positive, its impact on some vaccine serotypes remains controversial. As for other high-income countries that introduced PCV13 [6, 7, 23–25], in France, serotype 3 remained among the most frequent in older adults 4 years after PCV13 introduction, probably because of the absence of herd protection for this penicillin-susceptible serotype, which is rare in young children more frequently exposed to antibiotic consumption [26]. A recent study of the impact of PCV13 on PM cases in US children 3 years following vaccine introduction revealed that PM case numbers remain unchanged in the study sample and serotype 19A continued to be the most common serotype [27]. In our study, we observed a decrease of serotype 19A only in 2012–2013 in France. For this serotype the expected cross-protection from PCV7 because of the inclusion of the serogroup 19 by serotype 19F was limited and a delayed effect of the vaccine on this intrinsically more epidemic serotype, which was still in full expansion when the vaccine was introduced, was also reported in other countries [6, 28]. In the last few years, a resurgence of PCV7-serotype 19F was also observed, making it the most frequent serotype responsible for PCV7 failure in France [29]. This penicillin-resistant serotype might have been driven by high antibiotic exposure and vaccine-induced antibodies weaker than for the remaining serotypes [30].

Post PCV13 serotype replacement was highly variable across countries: in England and Wales an important increase of IPD due to non-PCV13-serotypes was observed (especially in children under 5 years old) [31]; in contrast, in other countries, such as Denmark, Spain, and the US, serotype replacement was limited, so that an overall decrease in IPD incidence was observed after the introduction of PCV13 [23, 25, 32]. In France, PCV13 favoured the emergence of other new emerging serotypes not included in the vaccine [16]. Our analysis of serotype distributions demonstrated that 12F, 24F, 23B and 10A were the most frequent non-vaccine serotypes that emerged in the entire French population during the post-PCV13 period. Serotype 12F reached the dominant position in 2011–2013 for the entire population and for children under 5 years old, thereby confirming earlier findings in France [33, 34] and other countries reporting its outbreak or hyperinvasivity [35–38]; its highly invasive disease potential was also recently shown in France for children under 2 years old [39]. With the same elevated invasive disease potential as 12F, we reported serotype 24F emergence in France [39], as in many other European countries [6, 40]. Despite the expected cross-protection between 6C and PCV13-serotype 6A [24, 41], we confirm a marked expansion of serotype 6C among adults above 64 years, previously observed in France [42] and other countries [6, 31].

Several limitations of this study should be noted. Due to the absence of a control group and limited pre-intervention data, a cause–effect relationship between the PCV introduction and PM evolution cannot be proved. Even though non-vaccine serotypes became dominant, other vaccine serotypes remained frequent, so vaccine impact cannot completely explain serotype evolution. Other factors, like specific natural competition, vaccination-induced cross-immunity, serotype-specific intrinsic epidemicity or periodic trend in serotype-colonisation turnover cannot be excluded [11, 25, 43, 44]. Furthermore, the selection of circulating pneumococcal strains can also be influenced by antibiotic exposure, which can interfere with the vaccine-induced serotype replacement. The lower intrinsic epidemic fitness of resistant pneumococcal strains [45] could explain why the antibiotic-susceptible strains are disadvantaged in a context of high level of antibiotic exposure but become more epidemic than resistant strains when antibiotic use declined. In France, the National campaign “Keep antibiotics working” led to a decrease of antibiotic exposure by at least 25% [2], the highest diminution among the developed nations, which has been suggested to have an impact on the incidence of severe infections caused by non-vaccine antibiotic-susceptible pneumococcal strains [5]. Because of lack of monthly data, we did not include antibiotic consumption series in our model.

## Conclusions

Vaccine preventable diseases like IPD require the development of an efficient surveillance system to monitor pre- and post-vaccination trends. In countries that have just introduced a new vaccine, the assessing of its impact is possible only if surveillance data had been collected both before and after vaccine introduction. Only an excellent surveillance system can allow to asses vaccine impact and therefore set up a future immunisation policy adequately. In countries with a settled immunisation program, because of the rapid ecologic modifications that we observed also in our analysis, a highly reactive surveillance system focusing on infections and colonisation is required. Public health decision-makers should be aware of the continuous evolution of the local PM epidemiology and they should demand that manufacturers rapidly adapt vaccines to the target population. Our study results highlight the emergence in France of a group of non-vaccine serotypes very different from those emerging in the US, especially among young children [27]. Despite the PCV15 vaccine covering an additional 21% of IPD cases among children in the US [23], in France, where 22F and 33F accounted for only 9% of PM cases in 2012–2014, this vaccine is not useful. If the observed non-vaccine serotype emergence continues a different formulation of a higher valent vaccine will be required.

## Additional file

Additional file 1: Table S1. Percentages (number) of isolated pneumococcal meningitis (PM) cases of the main pneumococcal serotypes, 2001–2014. **Figure S1** Total PM (grey) and model-estimated prediction (red) in children under 5 and adults above 64 years old, 2001–2014. **Figure S2.** Estimated PM cases percent changes compared to baseline (2001–2003) for adults above 64 years old. **Figure S3.** Number of PM cases occurring in adults above 64 years old according to serotype and penicillin susceptibility. (DOCX 751 kb)

## Abbreviations

IPD: invasive pneumococcal disease
NRCP: National Reference Center for Pneumococci
PCV13: 13-valent PCV
PCV7: 7-valent pneumococcal conjugate vaccine
PM: pneumococcal meningitis
PPV23: 23-valent polysaccharide pneumococcal vaccine
