# Supplementary material for: Impact of pneumococcal conjugate vaccines on pneumococcal meningitis cases in France between 2001 and 2014: a time series analysis
Source: BMC Med. 2016 Dec 21;14:211. doi: 10.1186/s12916-016-0755-7 (PMC5175381; doi:10.1186/s12916-016-0755-7)
Supplement: Additional file 1: Table S1. — Percentages (number) of isolated pneumococcal meningitis (PM) cases of the main pneumococcal serotypes, 2001–2014. Figure S1 Total PM (grey) and model-estimated prediction (red) in children under 5 and adults above 64 years old, 2001–2014. Figure S2. Estimated PM cases percent changes compared to baseline (2001–2003) for adults above 64 years old. Figure S3. Number of PM cases occurring in adults above 64 years old according to serotype and penicillin susceptibility. (DOCX 751 kb) [file 12916_2016_755_MOESM1_ESM.docx]

Additional file 1

Table S1…………………………………………………………………………………………………………………………………………………….……p. 2

Figure S1…………………………………………………………………………………………………………………………………………………….…...p. 3

Figure S2…………………………………………………………………………………………………………………………………………………….…...p. 4

Figure S3…………………………………………………………………………………………………………………………………………………….…...p. 5

***Table S1*. Percentages (number) of isolated pneumococcal meningitis (PMs) of the main pneumococcal serotypes, 2001-2014.**

| **PM** | **Epidemiological Period** | | | | | | | | | | | |
| --- | --- | --- | --- | --- | --- | --- | --- | --- | --- | --- | --- | --- |
| **Serotype** | **2001–2003** | **2003–2004** | **2004–2005** | **2005–2006** | **2006–2007** | **2007–2008** | **2008–2009** | **2009–2010** | **2010–2011** | **2011–2012** | **2012–2013** | **2013–2014** |
| PCV7 |  |  |  |  |  |  |  |  |  |  |  |  |
| 4 | 2% (17) | 3% (11) | 1% (6) | 2% (7) | 2% (9) | 2% (9) | 1% (5) | 0% (2) | 0% (1) | 1% (2) | 1% (2) | 1% (2) |
| 6B | 9% (61) | 9% (34) | 7% (29) | 7% (22) | 3% (12) | 2% (7) | 1% (6) | 1% (3) | 0% (2) | 1% (5) | 1% (3) | 0% (0) |
| 9V | 4% (29) | 7% (25) | 5% (19) | 4% (13) | 3% (10) | 2% (9) | 2% (8) | 1% (3) | 0% (0) | 0% (0) | 0% (0) | 0% (0) |
| 14 | 11% (82) | 7% (26) | 8% (31) | 5% (16) | 5% (21) | 4% (16) | 2% (8) | 1% (4) | 1% (3) | 1% (3) | 2% (6) | 0% (1) |
| 18C | 7% (49) | 6% (21) | 5% (21) | 7% (24) | 5% (21) | 5% (18) | 3% (12) | 0% (2) | 1% (6) | 2% (7) | 0% (0) | 0% (0) |
| 19F | 10% (70) | 10% (37) | 11% (43) | 9% (29) | 7% (28) | 5% (18) | 3% (15) | 2% (10) | 4% (18) | 4% (16) | 8% (28) | 6% (18) |
| 23F | 10% (73) | 9% (33) | 6% (24) | 5% (18) | 5% (19) | 3% (11) | 3% (13) | 1% (6) | 2% (7) | 1% (3) | 1% (3) | 0% (0) |
| Delta6^a^ |  |  |  |  |  |  |  |  |  |  |  |  |
| 1 | 1% (9) | 1% (3) | 2% (8) | 2% (8) | 2% (9) | 2% (9) | 2% (10) | 2% (9) | 1% (5) | 1% (3) | 1% (3) | 0% (1) |
| 5 | 0% (1) | 0% (1) | 0% (1) | 0% (0) | 0% (0) | 1% (2) | 0% (0) | 0% (0) | 0% (0) | 0% (0) | 0% (0) | 0% (0) |
| 7F | 2% (17) | 2% (7) | 3% (13) | 7% (24) | 8% (30) | 8% (28) | 13% (62) | 13% (55) | 7% (31) | 7% (25) | 3% (11) | 2% (5) |
| 3 | 6% (44) | 9% (31) | 8% (34) | 6% (20) | 7% (28) | 9% (31) | 9% (41) | 7% (29) | 3% (15) | 8% (30) | 6% (22) | 9% (26) |
| 6A | 5% (33) | 4% (13) | 3% (13) | 4% (13) | 4% (14) | 2% (7) | 3% (13) | 1% (6) | 1% (6) | 1% (4) | 0% (0) | 1% (3) |
| 19A | 7% (49) | 6% (21) | 6% (26) | 7% (22) | 12% (49) | 10% (35) | 14% (65) | 13% (54) | 14% (62) | 8% (28) | 5% (16) | 6% (18) |
| Delta2^b^ |  |  |  |  |  |  |  |  |  |  |  |  |
| 22F | 2% (11) | 3% (10) | 3% (11) | 3% (11) | 4% (15) | 4% (16) | 4% (21) | 5% (19) | 4% (17) | 4% (15) | 4% (14) | 5% (15) |
| 33F | 1% (7) | 0% (1) | 3% (13) | 3% (10) | 3% (13) | 1% (4) | 2% (9) | 2% (8) | 2% (10) | 2% (7) | 2% (8) | 3% (7) |
| Nonvaccine |  |  |  |  |  |  |  |  |  |  |  |  |
| 12F | 0% (1) | 0% (0) | 0% (0) | 0% (0) | 0% (0) | 1% (2) | 3% (14) | 8% (33) | 13% (58) | 12% (46) | 10% (34) | 7% (19) |
| 24F | 1% (8) | 2% (7) | 1% (4) | 4% (14) | 3% (12) | 7% (26) | 4% (18) | 3% (11) | 3% (13) | 5% (18) | 7% (25) | 7% (20) |
| 23B | 0% (3) | 0% (0) | 2% (8) | 1% (2) | 1% (2) | 3% (10) | 4% (18) | 4% (17) | 4% (18) | 2% (8) | 5% (17) | 8% (21) |
| 10A | 1% (6) | 2% (7) | 1% (5) | 3% (10) | 1% (4) | 3% (11) | 2% (8) | 3% (13) | 3% (11) | 4% (15) | 6% (20) | 6% (17) |
| 15A | 1% (10) | 2% (7) | 2% (8) | 0% (1) | 3% (13) | 2% (9) | 2% (11) | 5% (19) | 6% (28) | 6% (21) | 6% (20) | 4% (12) |
| 6C | 0% (0) | 2% (7) | 2% (7) | 1% (3) | 2% (8) | 1% (5) | 3% (12) | 5% (20) | 4% (18) | 8% (28) | 5% (17) | 4% (11) |
| 9N | 2% (17) | 2% (8) | 1% (5) | 2% (8) | 2% (7) | 1% (5) | 3% (13) | 2% (9) | 3% (11) | 1% (5) | 3% (9) | 5% (14) |
| 11A | 2% (17) | 1% (5) | 4% (15) | 2% (6) | 3% (10) | 3% (12) | 0% (2) | 2% (8) | 2% (8) | 2% (7) | 3% (11) | 3% (9) |
| 23A | 1% (4) | 1% (4) | 2% (7) | 2% (6) | 2% (7) | 1% (3) | 1% (6) | 2% (7) | 2% (7) | 2% (6) | 3% (10) | 3% (9) |
| 35B | 0% (0) | 0% (0) | 1% (3) | 2% (6) | 0% (7) | 0% (5) | 3% (14) | 5% (19) | 4% (18) | 3% (12) | 3% (10) | 2% (6) |
| 8 | 1% (10) | 2% (9) | 1% (6) | 1% (4) | 2% (8) | 2% (6) | 1% (3) | 2% (7) | 1% (6) | 1% (3) | 2% (6) | 3% (9) |
| 15C | 1% (6) | 2% (8) | 2% (10) | 2% (8) | 2% (6) | 2% (6) | 2% (8) | 1% (4) | 1% (4) | 2% (6) | 1% (5) | 3% (9) |
| 15B | 1% (8) | 1% (2) | 0% (2) | 2% (6) | 2% (7) | 3% (12) | 2% (9) | 2% (9) | 3% (13) | 3% (11) | 1% (5) | 1% (3) |
| 17F | 1% (5) | 1% (5) | 1% (5) | 1% (3) | 0% (0) | 2% (8) | 2% (8) | 2% (10) | 3% (14) | 2% (6) | 2% (6) | 1% (2) |
| 35F | 2% (15) | 2% (7) | 2% (9) | 2% (8) | 2% (8) | 1% (5) | 1% (7) | 1% (4) | 1% (4) | 1% (5) | 2% (6) | 0% (0) |
| 16F | 0% (1) | 0% (0) | 1% (5) | 1% (2) | 0% (1) | 1% (3) | 1% (7) | 0% (1) | 1% (5) | 1% (2) | 0% (1) | 1% (3) |
| Others | 7% (52) | 3% (12) | 4% (18) | 3% (9) | 4% (17) | 4% (15) | 6% (29) | 5% (20) | 3% (15) | 7% (25) | 9% (30) | 7% (20) |
| Total | 715 | 362 | 409 | 333 | 395 | 363 | 475 | 421 | 434 | 372 | 348 | 280 |

**^a^** Delta6 refers to the 6 serotypes added to PCV7 to obtain PCV13

^b^ Delta2 refers to the 2 serotypes added to PCV13 to obtain PCV15

***Figure S1*.** **Total Pneumococcal Meningitis (grey) and model-estimated prediction (red) in children <5 and adults >64, 2001-2014.**


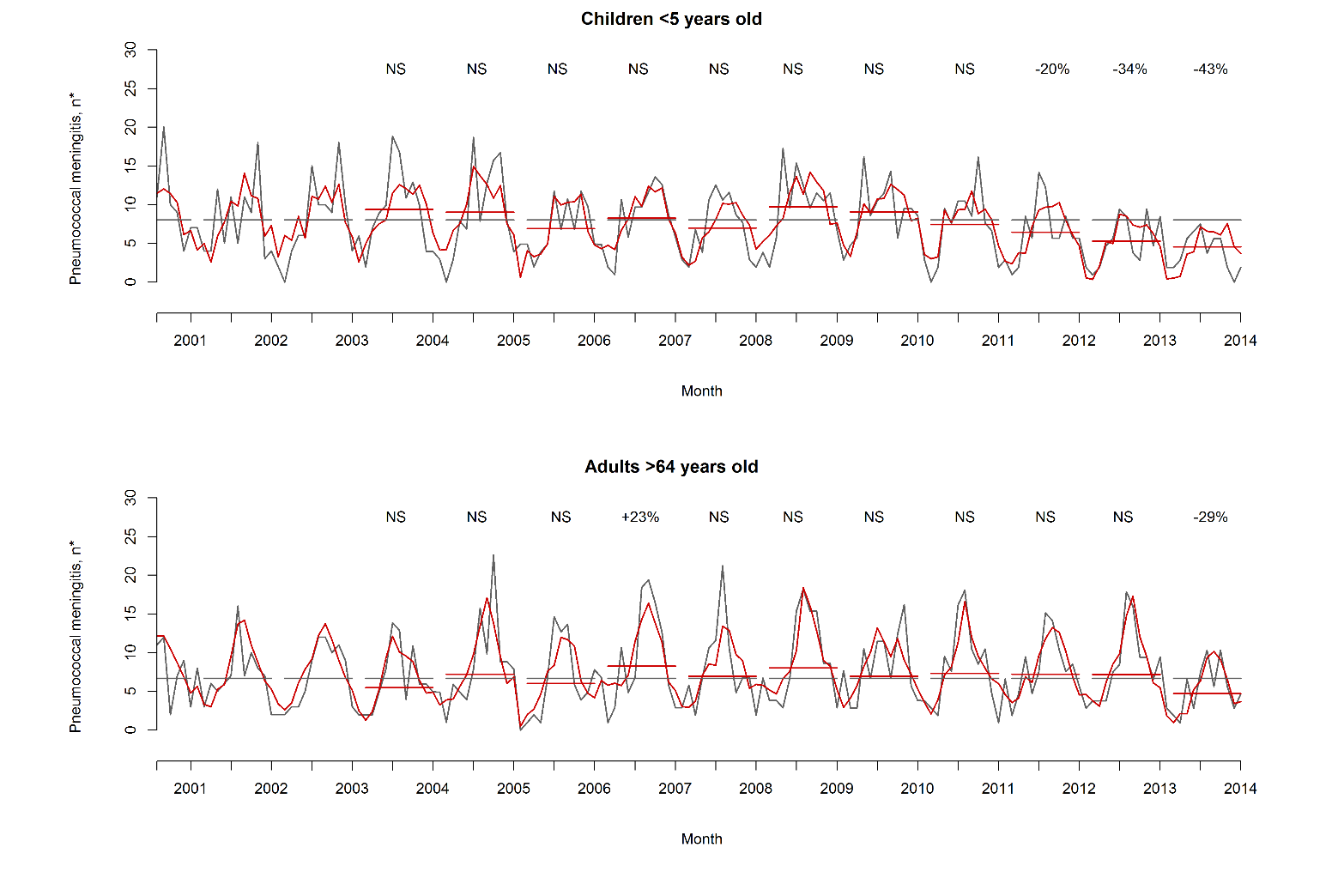


Grey lines are PMs expected during epidemiological periods under the assumption of no change since the baseline period (2001–2003). Red lines are PMs levels during epidemiological periods estimated by the model. The indicated percentages are significant relative changes compared to baseline values. NS: Non-significant. * Stable population.

***Figure S2*.** **Estimated PMs percent changes compared to baseline (2001–2003) for adults >64 years old.**


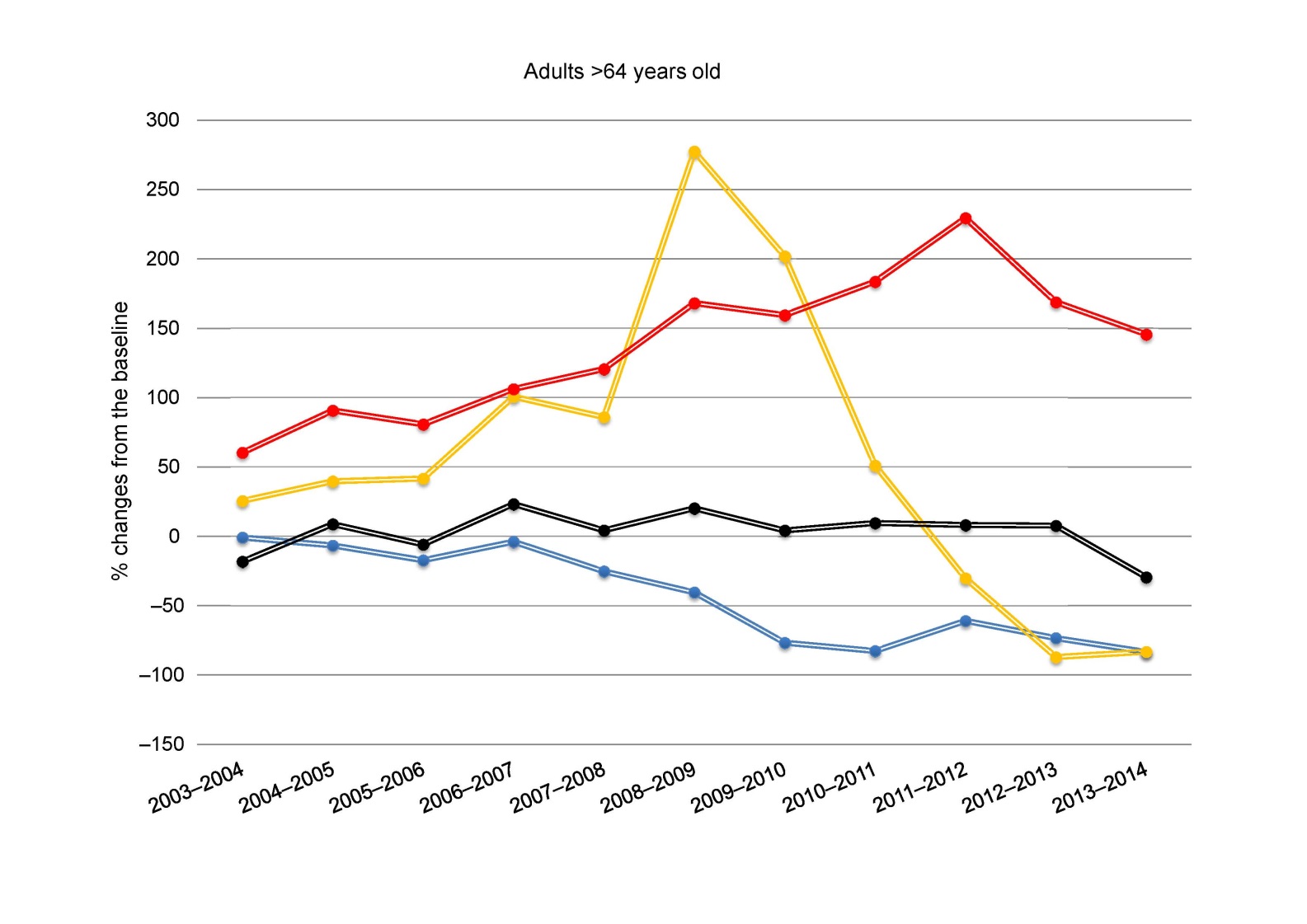


Total pneumococcal meningitis (PMs) (black), PCV7 (blue), the six added to PCV7 to obtain PCV13 (yellow) and non-vaccine (red) serotypes

***Figure S3*. Number of pneumococcal meningitis (PMs) occurring in adults >64 according to serotype and penicillin susceptibility.**


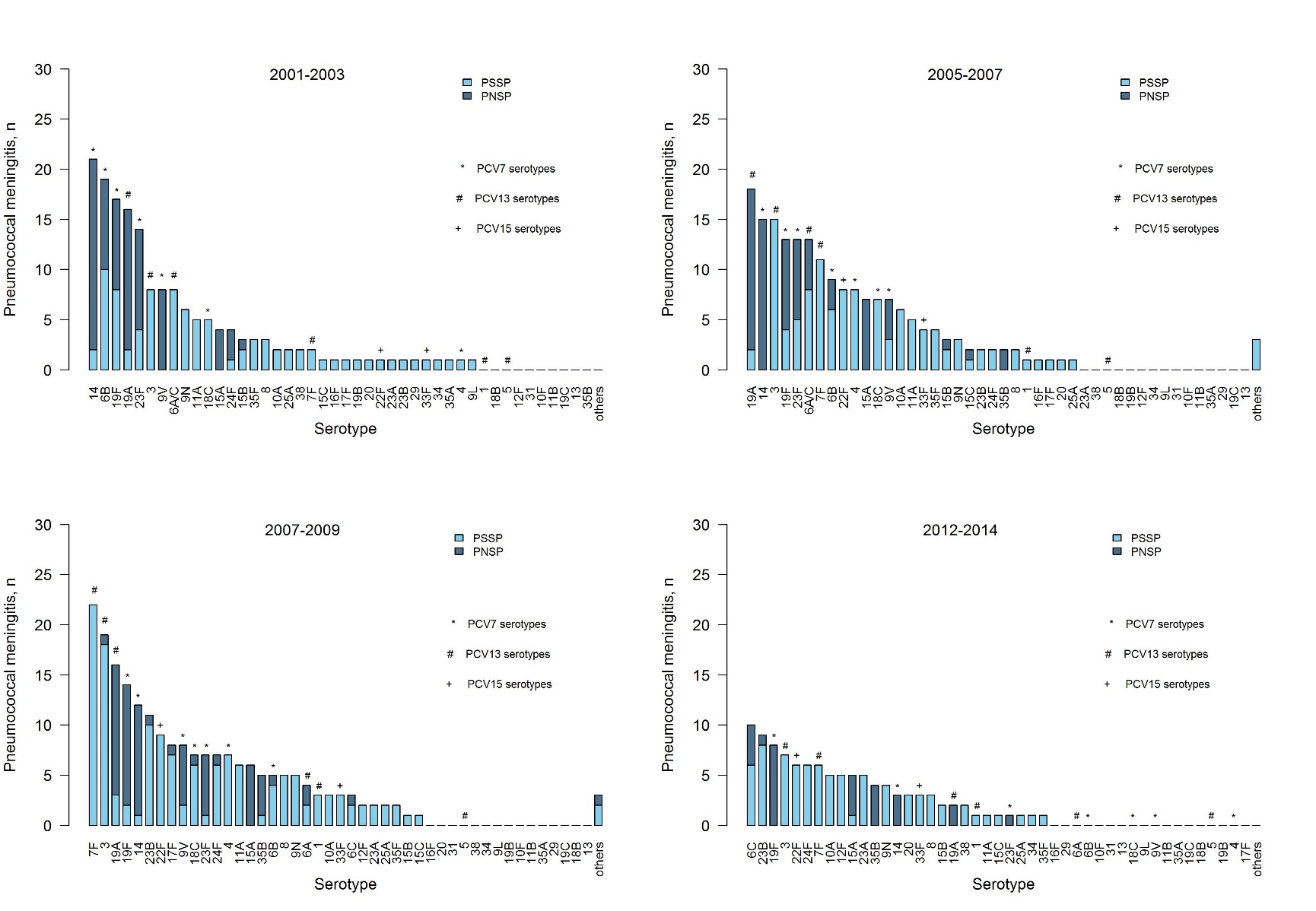


PSSP denotes penicillin-susceptible *Streptococcus pneumoniae* and PNSP penicillin-resistant *Streptococcus pneumoniae*.
